# Role of TGFβ signaling in the pathogenesis of Alzheimer’s disease

**DOI:** 10.3389/fncel.2015.00426

**Published:** 2015-10-28

**Authors:** Rommy von Bernhardi, Francisca Cornejo, Guillermo E. Parada, Jaime Eugenín

**Affiliations:** ^1^Laboratory of Neuroscience, Faculty of Medicine, Department of Neurology, Pontificia Universidad Católica de ChileSantiago, Chile; ^2^Laboratory of Neural Systems, Faculty of Chemistry and Biology, Department of Biology, Universidad de Santiago de ChileSantiago, Chile

**Keywords:** aging, cytokines, Glia, MAPK, NFκB, neurodegenerative diseases, neuroinflammation, transforming growth factor-β

## Abstract

Aging is the main risk factor for Alzheimer’s disease (AD); being associated with conspicuous changes on microglia activation. Aged microglia exhibit an increased expression of cytokines, exacerbated reactivity to various stimuli, oxidative stress, and reduced phagocytosis of β-amyloid (Aβ). Whereas normal inflammation is protective, it becomes dysregulated in the presence of a persistent stimulus, or in the context of an inflammatory environment, as observed in aging. Thus, neuroinflammation can be a self-perpetuating deleterious response, becoming a source of additional injury to host cells in neurodegenerative diseases. In aged individuals, although transforming growth factor β (TGFβ) is upregulated, its canonical Smad3 signaling is greatly reduced and neuroinflammation persists. This age-related Smad3 impairment reduces protective activation while facilitating cytotoxic activation of microglia through several cellular mechanisms, potentiating microglia-mediated neurodegeneration. Here, we critically discuss the role of TGFβ-Smad signaling on the cytotoxic activation of microglia and its relevance in the pathogenesis of AD. Other protective functions, such as phagocytosis, although observed in aged animals, are not further induced by inflammatory stimuli and TGFβ1. Analysis *in silico* revealed that increased expression of receptor scavenger receptor (SR)-A, involved in Aβ uptake and cell activation, by microglia exposed to TGFβ, through a Smad3-dependent mechanism could be mediated by transcriptional co-factors Smad2/3 over the MSR1 gene. We discuss that changes of TGFβ-mediated regulation could at least partially mediate age-associated microglia changes, and, together with other changes on inflammatory response, could result in the reduction of protective activation and the potentiation of cytotoxicity of microglia, resulting in the promotion of neurodegenerative diseases.

## Overview: Glial Cells and Neuroinflammation

Homeostasis of the nervous system is maintained by the finely tuned interaction of glial cells and neurons, involving a complex network of signaling pathways. Inflammation, a primarily beneficial process mediated by the activation of glia in response to injury, illness or infection, allows for the elimination of harmful stimuli and the repair of damaged tissue. However, this process can become dysregulated, when the activating stimulus cannot be removed, or in the context of a maintained inflammatory environment, as observed in aging (von Bernhardi et al., 2010). Thus, neuroinflammation can also be a self-perpetuating deleterious response, with persistent activation of glia, sustained release of inflammatory mediators, and increased oxidative and nitrosative stress; becoming a source of additional injury to host cells. Chronic neuroinflammation plays a role in a number of neurodegenerative diseases (Block and Hong, 2005; von Bernhardi et al., 2007; Gao and Hong, 2008), inducing neuronal injury.

Microglia are the brain resident innate immune system (Hemmer et al., 2002; Ransohoff and Perry, 2009; Rivest, 2009). When stimulated, microglia activate and change their functional properties (Liu et al., 2001; von Bernhardi and Eugenin, 2004; Lue et al., 2010). They can sense and respond to a wide range of stimuli, including central nervous system (CNS) trauma, ischemia, infection, toxic, and autoimmune insults (Kreutzberg, 1996; Streit, 2002; Kim and de Vellis, 2005; Schwab and McGeer, 2008; Lue et al., 2010; von Bernhardi et al., 2010). In fact, microglia are activated in virtually all CNS diseases (Kreutzberg, 1996; Hanisch and Kettenmann, 2007; Neumann et al., 2009). They are the main producers of a broad spectrum of inflammatory mediators, such as eicosanoids, cytokines (Nakamura, 2002; Kim and de Vellis, 2005; Tichauer et al., 2007), chemokines, reactive oxygen species (ROS), nitric oxide (NO)·, small metabolite mediators, and proteases (α-antichymotrypsin and α-antitrypsin) (Benveniste et al., 2001; Nakamura, 2002; Streit, 2002; Li et al., 2007; Tichauer et al., 2007; Neumann et al., 2009; Lue et al., 2010).

Other glial cell type, astrocytes, which will not be discussed in this work, share several functions with microglia. They are important for neurotrophic support and metabolism, synaptic regulation and several other functions, in addition to their participation in β-amyloid (Aβ) *clearance* (Rossner et al., 2005; Murgas et al., 2012). Inflammatory mediators regulate the innate immune defense, induce bystander damage, and modify synaptic function (Aldskogius et al., 1999; Selkoe, 2002; Di Filippo et al., 2008) according to environmental conditions (Li et al., 2007; von Bernhardi, 2007). Depending on the activation context, microglia secrete inflammatory cytokines such as interleukin 1β (IL1β), tumor necrosis factor α (TNFα) and interferon gamma (IFNγ), and reactive species (Kettenmann et al., 2011), as well as regulatory cytokines like interleukin 10 (IL10) and transforming growth factor β (TGFβ1; Nakajima et al., 2007; Sierra et al., 2007; Welser-Alves and Milner, 2013). Inflammatory cytokines trigger the production of several inflammatory factors that could affect neuronal function. In response to IFNγ, for example, glia produce NO· by up-regulation of inducible nitric oxide synthase (iNOS) and release superoxide radicals (O_2_·^−^) by a nicotinamide adenine dinucleotide phosphate (NADPH)-oxidase mediated mechanism (Hu et al., 1995; Calabrese et al., 2007). Neuroinflammation affects neuron-glia crosstalk and establishes interactions with oxidizing agents through redox sensors in enzymes, receptors, and transcription factors, all of which can affect neuronal function (Liu et al., 2012), inducting neurodegeneration (Raj et al., 2014). Oxidative stress, in turn, further increases inflammatory cytokines, creating a vicious cycle (Rosales-Corral et al., 2010), with profound impact in cell homeostasis and survival (Satoh and Lipton, 2007).

Astrocyte and microglia activation occur through the phosphorylation of MAPKs and the activation of nuclear factor *kappa* B (NFκB) pathway, inducing the expression of inflammatory mediators (Van Eldik et al., 2007; Glass et al., 2010; Heneka et al., 2010). MAPKs include extracellular signal-regulated protein kinases (ERKs) and stress activated protein kinases c-Jun NH2-terminal kinase (JNK) and P38. Activated MAPKs exert their actions both in the cytoplasm and translocating into the nucleus, phosphorylating transcription factors. Noteworthy, ERK and P38 appear to be key actors in the production of free radicals (Bhat et al., 1998; Marcus et al., 2003; Qian et al., 2008). The ERK pathway is regulated by pro- and anti-inflammatory cytokines, determining the timing of microglia activation (Saud et al., 2005; Glass et al., 2010). In addition, P38 is involved in the production of NO· by up-regulating iNOS (Saha et al., 2007; Munoz and Ammit, 2010), and enhances the expression of inflammatory cytokines, such as TNFα, through both transcriptional and post-transcriptional mechanisms. P38 can induce transcription of the TNFα gene by increasing activator protein-1 (AP-1) activity (Spriggs et al., 1992) and enhances its production by increasing the stability and translation of TNFα mRNA (Dean et al., 2004).

Activation of microglia shows a broad functional spectrum associated with specific expression patterns of cytokines and their receptors (Town et al., 2005). Depending on the stimuli they receive, they show different activation profiles (Gordon, 2003; Martinez et al., 2008; Mosser and Edwards, 2008), including: (i) classical activation (M1 activation), which under certain conditions will be cytotoxic; (ii) alternative phagocytic/neuroprotective (M2 activation; Gordon, 2003; Martinez et al., 2008); or (iii) regulatory activation (Mosser and Edwards, 2008). Activation of interferon-regulatory factor 5 (IRF5), defines commitment to the M1 macrophage lineage (Satoh et al., 2010), while IRF4 controls M2 polarization (Satoh et al., 2010; Krausgruber et al., 2011). In M2 macrophages, activation of NFκB-p50 appears to be associated with the inhibition of M1-polarizing genes (Porta et al., 2009). M2-type induction, through secretion of IL4, IL10 and TGFβ, promotes humoral immune responses and down-regulates M1 responses, inhibiting many macrophage inflammatory functions (Town et al., 2005). A third group, regulatory macrophages, arises at later stages and have a primary role limiting inflammation (Mosser, 2003; Lucas et al., 2005; Mosser and Edwards, 2008).

## Age-Related Changes of Microglial Cells

The term “inflamm-aging” was coined in reference to the state of mild chronic inflammation (Franceschi et al., 2000) observed in aged individuals, functionally characterized by a reduced capability to deal with stressing stimuli. The age-related immune changes, known as immune-senescence (Larbi et al., 2008), would be also induced by cumulative low-level inflammation, which induces changes in gene expression related to inflammation and immune response (Lee et al., 2000; de Magalhães et al., 2009), increases plasmatic levels of inflammatory cytokines (Singh and Newman, 2011), and activates inflammatory intracellular pathways (Helenius et al., 1996).

**Figure 1:**
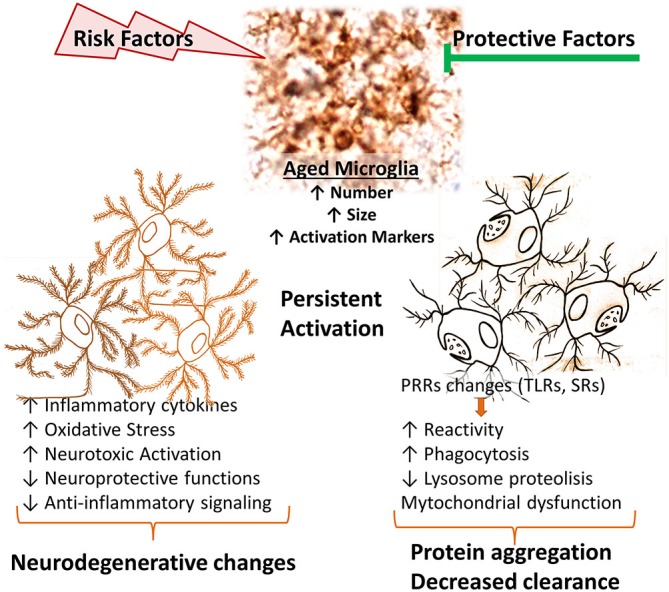
**Age-related changes of microglial cell function.** In aged brains, there is an increased number, size and activation of microglia. Age-related microglia changes depend both on gained and lost functions. Diverse stimuli or injury processes can further promote an inflammatory environment, promoting cytotoxic microglial cell activation. Aged microglia show increased basal phagocytic activity, although a reduced capacity to induce phagocytosis when stimulated, together with reduced lysosomal activity, resulting in a decreased clearance activity. They also have increased production of inflammatory cytokines and reactive species. Those changes result in a shift of balance towards decreased protective functions and an increased neurotoxicity. PRRs, pattern recognition receptors; SRs, scavenger receptors; TLRs, Toll-like receptors.

In aged animals, protein homeostasis is impaired at multiple levels, including chaperone-mediated protein folding and stability, protein trafficking, protein degradation and autophagy. A major consequence of these impairments is the aggregation of abnormal proteins, which are related to neurodegenerative diseases, such as Parkinson’s disease (PD) and Alzheimer’s disease (AD; Taylor and Dillin, 2011). Aged microglia undergo multiple functional changes (reviewed in von Bernhardi et al., 2015), impacting the neuronal environment and promoting development of cognitive impairments (Figure 1). Among these changes, microglial cell production of ROS and inflammatory cytokines could contribute to the onset of chronic neurodegenerative diseases (von Bernhardi, 2010). Decline of lysosomal and mitochondrial functions results in an exacerbated generation of ROS and inflammatory mediators by microglia. Moreover, aged microglia show a decreased ability to phagocytose Aβ in comparison with young microglia (Floden and Combs, 2011).

In aging, activated microglia remain as the principal source of inflammatory molecules and oxidative products of the CNS (Pawate et al., 2004; Qin et al., 2005; Hayashi et al., 2008). Both basal production of IL-6 and lipopolysaccharide (LPS)-induced secretion of IL-6 and IL1β are higher in aged microglia than in younger cells (Ye and Johnson, 1999; Sierra et al., 2007). In fact, mild stimulatory events or minor injuries, otherwise innocuous, could induce a robust and potentially damaging response. Thus, a stimulus that normally would trigger a protective response, in presence of age-related impairment of regulation could determine a persistent activation, associated, for example, to oxidative stress (von Bernhardi, 2007; Herrup, 2010). Similarly, aged microglia become more inflammatory than their younger counterparts upon systemic inflammatory stimulation, exacerbating damage (Combrinck et al., 2002; Cunningham et al., 2005; Godbout et al., 2005; Sierra et al., 2007). Accordingly, when exposed to endotoxin, microglia derived from adult mice secrete high amounts of ROS, whereas those from young animals mostly produce NO (Tichauer et al., 2014). Those effects depend, at least partly, on the upregulation of Toll-like receptors (TLRs), increased expression of the TLR4 co-receptor CD14 (Letiembre et al., 2007), changes in TLR4 signaling, and changes on the expression profile of scavenger receptors (SRs; Yamamoto et al., 2002; Hickman et al., 2008).

The activation of TLRs, CD14, and SRs by specific ligands is associated with microglial cell activation (Godoy et al., 2012; Murgas et al., 2012; Nakajima et al., 2007), production of inflammatory mediators, and uptake of macromolecules, including Aβ (Alarcón et al., 2005). There is conflicting evidence regarding the effect of age on phagocytosis. In contrast with reports indicating that microglia from aged mice have a decreased ability to phagocytose Aβ compared with young mice (Floden and Combs, 2011), we observed that basal phagocytosis of aged microglia is slightly increased compared with that from young mice, but phagocytosis fails to be induced by TGFβ (Tichauer et al., 2014) or LPS (Cornejo et al., 2014). Class A SR (SR-A) appears to play a key role for Aβ internalization by microglia (Chung et al., 2001) and degradation by cathepsin B (Yang et al., 2011), and for activation of microglia (Cornejo and Von Bernhardi, 2013). SR-A participates in the phagocytosis of Aβ and other anionic molecules, leading to the production of ROS (El Khoury et al., 1996). In AD, microglia expressing SR-A have been observed in close association with senile plaques (Honda et al., 1998; Bornemann et al., 2001). SR-A inhibition appears to increase Aβ burden in the brain of AD patients, potentially promoting neurotoxic effects and disease progression (Frenkel et al., 2013). The expression of these receptors decreases in the brain of aging animal models of AD (Hickman et al., 2008). Age-related changes in the expression of receptors involved in inflammatory activation could account for part of the function impairment of microglia, and provide insight regarding cell phenotypes that could play a role in the pathophysiological changes leading to neurodegenerative diseases. Given the various protective functions served by microglia, rather than seeking the inhibition of microglia, the regulation of those receptors involved in microglia activation could reduce some of the deleterious effects secondary to age-related microglial cell dysfunction.

## Microglia and Alzheimer’s Disease

Neuropathology in AD is characterized by the deposition of Aβ plaques and neurofibrillary tangles, constituted by hyper-phosphorylated tau, in the brain parenchyma (Hardy and Selkoe, 2002), intimately associated with activated microglia and astrocytes (Kim and de Vellis, 2005; Jellinger, 2006; Heneka and O’banion, 2007; von Bernhardi, 2007; von Bernhardi et al., 2010), and loss of synapses and neurons (Uylings and de Brabander, 2002). Worth mentioning, Aloise Alzheimer already stated in the early 1900’s that plaques and tangles probably were markers of an upstream process rather than the disease cause (Davis and Chisholm, 1999).

Microglial cell reactivity to Aβ and phagocytic activity are modulated by astrocytes, attenuating the cytotoxic response of microglia (DeWitt et al., 1998; von Bernhardi and Ramírez, 2001). However, modulation is abolished when microglia exposed to Aβ was previously primed (von Bernhardi and Eugenin, 2004), condition in which microglia show increased cytotoxicity, Aβ precursor protein (APP) synthesis, Aβ aggregation, and impairment of the uptake and degradation of Aβ compared with non-activated microglia (Rogers et al., 2002; von Bernhardi et al., 2007; Ramírez et al., 2008).

P38 appears to be involved in several pathological processes of AD. P38 becomes activated at early stages of the disease (Pei et al., 2001; Sun et al., 2003), being one of the kinases that phosphorylates specific sites in tau (Feijoo et al., 2005; Churcher, 2006). Inhibition of P38 abolishes Aβ-induced neuronal death *in vitro* (Zhu et al., 2005). P38 and NFκB appear to have a critical role for glial cell activation. Activation of those pathways are involved in Aβ-mediated induction of NO and TNFα production by glia (O’Neill and Kaltschmidt, 1997; Akama et al., 1998; Saha et al., 2007; Munoz and Ammit, 2010), which correlates with Aβ-induced cognitive impairment (Tran et al., 2001; Wang et al., 2005; He et al., 2007; Medeiros et al., 2007). Stimulation by Aβ induces a transient phosphorylation of P38, and a slower activation of NFκB (Flores and von Bernhardi, 2012) depending on the up-regulation of the transcriptional activity of NFκB by P38 (Saha et al., 2007), which contributes to neuroinflammation by activating AP-1 and by stabilizing mRNA and enhancing activity of NFκB. Production of TNFα and NO have different temporal profiles, in agreement with the early induction of cytokines by Aβ that appears to be needed for the subsequent induction of iNOS expression (Akama and Van Eldik, 2000).

### The “Glial Dysfunction Hypothesis”

**Figure 2:**
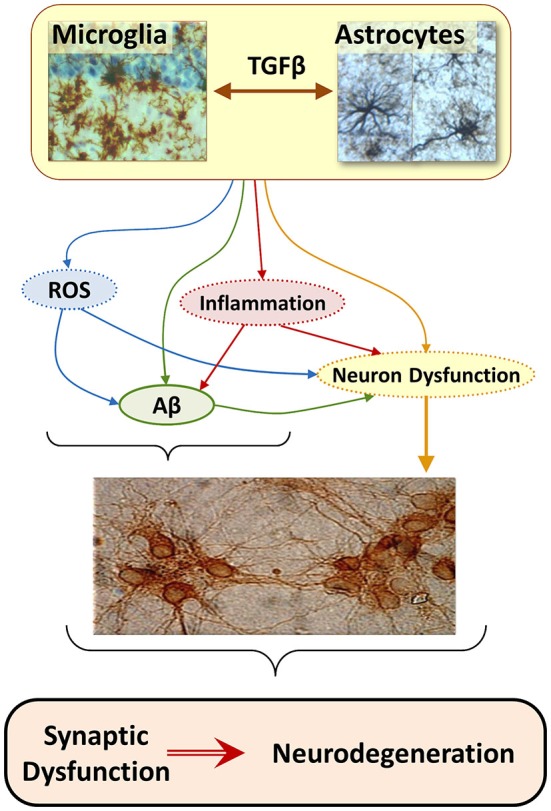
**The “Glial Cell Dysregulation Hypothesis” for Alzheimer’s disease (AD).** The glial cell dysregulation hypothesis proposes that AD has its cause on changes on the activation and impaired regulation of microglia, which become increasingly cytotoxic decreasing their protective functions. Microglia is under the regulation of astrocytes which, among other factors, secrete TGFβ. Inflammatory activation, secondary to aging and to certain forms of pathological stimuli, can result in glial cell dysregulation. Dysregulated glia, though the abnormal release of cytokines, reactive species, and other mediators, contributes to the increased expression of Aβ precursor protein (APP) and aggregation of Aβ, as well to functional and degenerative changes of neurons, perpetuating abnormal activation of glia, synaptic dysfunction and cell damage.

The consideration that brain innate immune response can be involved in the genesis of neurodegenerative diseases (Nguyen et al., 2002; Bjorkqvist et al., 2009; von Bernhardi et al., 2010), lead to re-consider the role of Aβ and propose glia as a leading factor in AD pathology (Figure 2; von Bernhardi, 2007). However, for most scientists who adhere to the “amyloid cascade hypothesis”, Aβ is viewed as the cause of AD and neuroinflammation is considered just a consequence of glia activation (Akiyama et al., 2000; Heneka and O’banion, 2007; Hirsch and Hunot, 2009).

Astrocytes modulate microglia cytotoxicity and phagocytosis of Aβ (von Bernhardi and Ramírez, 2001). TGFβ1, secreted by astrocytes and neurons among other cells, regulates microglia activation, reducing release of inflammatory cytokines and reactive species (Chen et al., 2002; Mittaud et al., 2002; Herrera-Molina and Von Bernhardi, 2005; Herrera-Molina et al., 2012), protecting neuronal cells *in vitro* (Hu et al., 1995; Lieb et al., 2003; Herrera-Molina and Von Bernhardi, 2005) and promoting phagocytosis (Wyss-Coray et al., 2001). However, chronically activated microglia show a reduced response to such a modulation (von Bernhardi and Eugenin, 2004), showing instead an increased cytotoxicity and impaired uptake of Aβ (von Bernhardi et al., 2007; Ramírez et al., 2008). Regulation by TGFβ1 depends on a Smad3-mediated mechanism (Flores and von Bernhardi, 2012; Tichauer and von Bernhardi, 2012). Age-related inhibition on the activation of Smad has a profound effect on the regulation of microglia by TGFβ (Tichauer et al., 2014).

In the context of the “glial cell dysregulation hypothesis” neurotoxicity is not viewed as a consequence of hyperactive but rather of “mis-active”, dysfunctional microglia (von Bernhardi, 2007). Solid evidence show that adequately activated microglia are needed as scavenger cells in the CNS, participating for example in Aβ clearance (Paresce et al., 1996; Alarcón et al., 2005). However, lost response to normal regulatory feedback and/or an impaired ability to clear Aβ (Paresce et al., 1997; von Bernhardi, 2007), could lead microglia to develop predominantly cytotoxic features, establishing an inflammatory environment with increased oxidative stress, conditions that are amyloidogenic (Gabuzda et al., 1994; Wang et al., 2004), and promote neuron dysfunction (Figure 2). Thus, microglia, initially protective, would become chronically activated and show an exacerbated reactivity, contributing to brain cytotoxicity and neurodegeneration (Nguyen et al., 2002; Wyss-Coray and Mucke, 2002; Saud et al., 2005).

Regulation of glial cell activation appears to be impaired under sustained inflammatory stimulation (Ramírez et al., 2008), as those observed in the aged brain (Tichauer et al., 2014). Whereas inflammatory activation of glia by Aβ is relatively mild in culture, it is markedly potentiated in primed glia (von Bernhardi et al., 2007). Likewise, attenuation of microglia reactivity by astrocytes is greatly reduced when glia are exposed to inflammatory conditions (von Bernhardi and Eugenin, 2004). The priming of glia, rather than Aβ, could be the main trigger for abnormal glia activation in response to a stimulus that normally would not produce a sustained robust activation, a condition we named “dysregulated glia” (von Bernhardi, 2007). In that sense, in contrast to microglia normally reacting mildly when exposed to Aβ, microglia have an enhanced activation under chronic inflammatory conditions. Enhanced activation in turn could result in an increased cytotoxicity (von Bernhardi et al., 2015).

## Role of TGFβ in the “Glial Cell Dysregulation Hypothesis”

**Figure 3:**
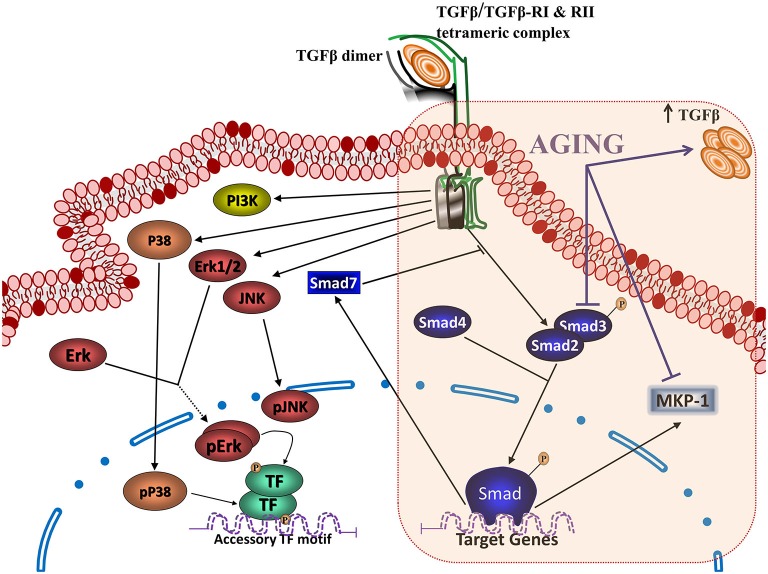
**Transforming growth factor β (TGFβ) signaling pathways and aging.** Binding of TGFβ to the type II TGFβ receptor dimer (TGFβRII) triggers recruitment of type I receptor dimer (TGFβRI), generating the heterotetrameric TGFβ receptor capable of activating the intracellular signaling pathways for TGFβ action. The activation of this complex activates (a) the canonical TGFβ signaling, with the phosphorylation of Smads 2 and/or 3 dimers, which bind to Smad 4 and translocate into the nucleus to regulate gene transcription, together with the activation of (b) non-canonical TGFβ signaling, which includes activation of MAPKs (ERK, extracellular signal-regulated protein kinase; JNK, c-Jun NH2-terminal kinase; and P38) and PI3K. Aging results in several changes on TGFβ signaling, including an increased production of TGFβ, as well as inhibition of the Smad pathway and activation of the phosphatase MAPK phosphatase (MKP-1).

TGFβ is present in three isoforms, TGFβ1, TGFβ2 and TGFβ3. Astrocytes secrete preferentially TGFβ1. Increased production of TGFβ1 in response to inflammatory conditions is one of the regulatory mechanisms secondary to cell activation (Herrera-Molina and Von Bernhardi, 2005) that limits the temporal and spatial extent of neuroinflammation (Ramírez et al., 2005; Saud et al., 2005), and neurotoxicity (Eyupoglu et al., 2003). The modulation exerted by TGFβ1 is mediated by the activation of Smad3, which is down regulated in AD patients (Colangelo et al., 2002) and aged mice (Tichauer et al., 2014), and the activation of ERK (Saud et al., 2005), which also appears to be neuroprotective under certain conditions (Zhu et al., 2002, 2004). In addition to Smad, dynamic regulation of PI3K and MAPK, which are activated as part of the TGFβ signaling pathway (Figure 3) as well as with other inflammatory cytokines, can be key factors for cell viability and the regulation of inflammation. In the following sections, we will discuss how inhibition of Smad3 pathway and increased levels of TGFβ, as observed in aging, could modify regulatory signals, leading to glia dysregulation. The lack of inhibition of microglia inflammatory activation by TGFβ could result in cytotoxicity and neurodegenerative changes as those observed in AD. Impairment of TGFβ-Smad3 signaling could reduce the capability of microglia to deal with injury, inhibiting beneficial responses while inducing progression towards a more inflammatory state in the aging brain (Franceschi et al., 2000). This neuroinflammatory state could favor the development of age-related neurodegenerative diseases (Larbi et al., 2008), affecting the regulation of several inflammatory signaling pathways as we will discuss on the following sections.

## TGFβ Signaling Pathways and Their Regulation

TGFβ1 is a pleiotropic cytokine and a potent regulator of neuroinflammation and cytotoxicity. Many beneficial effects depend on the regulation of microglial cell activity by TGFβ1 (Hu et al., 1995; Lieb et al., 2003; Herrera-Molina and Von Bernhardi, 2005). In the brain, TGFβ1 is associated with neuroprotection in excitotoxicity, hypoxia, and ischemia, as well as with interfering with cell death cascades induced by compounds such as Aβ (Caraci et al., 2011). TGFβ1 secreted at the injury site promotes microglia recruitment, allowing for an efficient removal of the noxious stimulus. Stimulation of hippocampal cultures with LPS and IFNγ increases the secretion and activation of TGFβ1 (Uribe-San Martén et al., 2009). TGFβ1 secreted by hippocampal neurons and astrocytes *in vitro* (Ramírez et al., 2005; Tichauer et al., 2007) and microglia (Welser-Alves and Milner, 2013) decreases release of inflammatory mediators, ${\text{O}}_{2}^{-}$ and NO by microglia (Chen et al., 2002; Mittaud et al., 2002; Herrera-Molina and Von Bernhardi, 2005; Saud et al., 2005; Herrera-Molina et al., 2012), and increases viability of neurons (Hu et al., 1995; Lieb et al., 2003; Herrera-Molina and Von Bernhardi, 2005). Inhibition of LPS-induced macrophage and microglia activation by TGFβ1 is regulated in a Smad3-dependent manner (Werner et al., 2000; Le et al., 2004). The same mechanism is also involved in astrocyte-mediated neuroprotection against N-methyl-D-aspartate (NMDA)-induced neuronal injury (Docagne et al., 2002; Katayama et al., 2010) that results from the sustained activation of the ERK pathway, playing also a pivotal role in astrogliosis (Chu et al., 2004).

TGFβ signaling pathway (Figure 3) is activated when TGFβ interacts and induces assembly of an heterotetrameric receptor, containing two serine/threonine kinase receptors, type II and type I (Rahimi and Leof, 2007). In mammals, there are five type II receptors, TβRII, ActR-II, ActR-IIB, BMPR-II, AMHR-II, and seven type I receptors, the *activin receptor-like kinases 1-7 (ALK1-7*; Rahimi and Leof, 2007). In canonical TGFβ signaling pathway, ligand binding induces type II receptor to phosphorylate and activate type I receptor, which then phosphorylates receptor activated members of the Smad family (*R-Smad*). TGFβ activates the phosphorylation of Smad2 and Smad3, their assembly with a Smad common-partner, Smad4, and the nuclear translocation of the heterotrimeric complex (Smad2/Smad2/Smad4, Smad3/Smad3/Smad4 or Smad2/Smad3/Smad4). In the nucleus, the complex interacts with AGAC enriched Smad binding elements (SBE) on the DNA, acting as a transcriptional co-activator (Wrighton et al., 2009). They bind to specific sequences where they can activate or inhibit transcription, regulating gene expression of target genes associated with inflammatory activation (Schmierer and Hill, 2007; Heldin and Moustakas, 2012), including that of Smad 7 (Ross and Hill, 2008) that belongs to a third type, the inhibitory Smads (*I-Smads*: Smad6/7), which are an endogenous inhibitory system.

Smad pathways act as co-factors coupled to master transcriptional factors to direct gene expression in a cell-specific manner (Mullen et al., 2011). The main regulation of TGFβ signaling occurs on Smad. For example, Smad2/3 can be acetylated by transcriptional co-activators such as p300 and CREB-binding protein (CBP) in a TGFβ-dependent way (Simonsson et al., 2006; Tu and Luo, 2007). This post-transduction modification favors binding of the Smad complex to DNA, and the transcription of its target genes (Simonsson et al., 2006). Also, phosphorylation of Smad3 mediated by CDK2 and CDK4 inhibits its transcriptional activity (Liu, 2006; Buxton and Duan, 2008), and Smad3-PIAS (*protein inhibitor of activated STAT*) interaction suppresses Smad3 activation by TGFβ and favors its SUMOilation (Imoto et al., 2003). These and others post-transduction modifications, such as MAPKs-mediated phosphorylation (see below), can also regulate Smad activity and therefore TGFβ-mediated transcription (Ross and Hill, 2008).

In addition to TGFβ-mediated Smad activation, TGFβ activates a complex Smad-independent (TGFβ-non canonical pathways) signaling pathway (Weiss and Attisano, 2013; Figure 3). Non canonical signaling includes MAPK pathways ERK, P38 and JNK, and PI3K/Akt (Derynck and Zhang, 2003; Weiss and Attisano, 2013), and participate in many biological processes such as cell cycle inhibition, immunosuppression and neuroprotection, among others (Bosco et al., 2013).

## Regulation of Inflammation-Related Genes by TGFβ

TGFβ orchestrates the expression of numerous genes associated with inflammation and the immune response. In the nervous system, the TGFβ pathway is involved in the regulation of genes associated with cell cycle, cell proliferation, preservation of neural progenitor cells, oligodendroglia and neuronal differentiation, neuron survival and function, and the several neurotransmission-related genes (Kandasamy et al., 2014). TGFβ has a role in adult neurogenesis (He et al., 2007), and in the differentiation of adult neural progenitor cells, inducing the expression of several voltage-dependent channel subunits (Kcnd3, Scn1b, Cacng4, and Accn1) and other neuronal proteins, such as Cadps, Snap25, Grik4, Gria3, Syngr3, Gria4, doublecortin (DCX), Nrxn1, Sept8, and Als2cr3, suggesting that TGFβ participates, at least in part, in the induction of a functional neuronal phenotype, (Kraus et al., 2013). In addition, TGFβ participates in cell migration by modulating the expression of cell adhesion proteins such as nCAM, integrin α3, αV and β1 (Siegenthaler and Miller, 2004; Milner, 2009).

TGFβ regulates MHC class II expression in astrocytes, (Johns et al., 1992). It also regulates the expression of some constituents of its own pathway, including receptors type I TGFβ receptor (TGFβRI) and type II TGFβ receptor (TGFβRII), Smad7 and Smad3 (Ma et al., 2007; Qin et al., 2009). Microglia treated with TGFβ show a reduced expression of the immune mediators CCL3, CCL2, IL1a, IL1rl2, CCR5 and CD11c (Abutbul et al., 2012; Butovsky et al., 2014), and upregulation of CX3CR1, CSF3 and TLR3 expression (Chen et al., 2002; Butovsky et al., 2014), as well as SRs, which will be discussed in a later section.

## Age-Related Changes on the TGFβ Pathway

In the brain, TGFβ favors cell survival, modulating the expression of Bad, Blc-2 and Bcl-x1 as a mechanism of neuroprotection against apoptosis (Dhandapani and Brann, 2003). TGFβ also participates in the regulation of temporal transition between early and late phases of neurogenesis and the regulation of the stem cell potency (Dias et al., 2014). Increased levels of TGFβ have been reported in several brain areas, including the hippocampus and hypothalamus, during aging (Bye et al., 2001; Werry et al., 2010). Cells showing increased production of TGFβ apparently do not include neurons, since TGFβ transcripts level are severely reduced in aged neurons (de Sampaio e Spohr et al., 2002).

Aging also affects the circadian variation of TGFβ expression. It has been reported a loss of the diurnal pattern of TGFβ expression, as well as a loss of the day/night expression of activated Smad3 compared with the pattern observed in young animals (Beynon et al., 2009) that have profound functional effects. In addition, the pedunculopontine (PPT) nucleus of aged rats, a structure related to sleep and cognitive functions, shows an over activation of the TGFβ-Smad signaling pathway that appears to be involved in sleep-related memory impairment in aging (George et al., 2006).

There are age-related changes on TGFβ signaling at several levels. Some depend on changes on the level or the pattern of secretion of TGFβ, or in its canonical signaling pathway, Smad (Figure 3). Others depend on changes on the interaction of TGFβ with other inflammatory mediators or their transcription factors, such as IFNγ and NFκB, or on regulatory components, such as MAPK phosphatases (MKP-1). Finally, there are age-related changes on its regulation on cellular processes, as observed with stem cells. The various changes will be discussed on the following sections.

### Aging-Related Inhibition of the TGFβ-Smad Pathway

TGFβ1, produced by astrocytes *in vitro*, decreases microglial NO and ROS production induced by LPS and IFNγ (Herrera-Molina and Von Bernhardi, 2005). Induction of both NO and ROS is prevented by TGFβ1 in neonatal, but not in adult animals. Therefore, response to inflammatory stimulation appears to become more oxidative, and for that reason, potentially more cytotoxic in aged animals (Tichauer et al., 2014). Moreover, modulation by TGFβ1 also is abolished in microglia obtained from animals previously exposed to inflammatory conditions (Tichauer et al., 2014).

**Figure 4:**
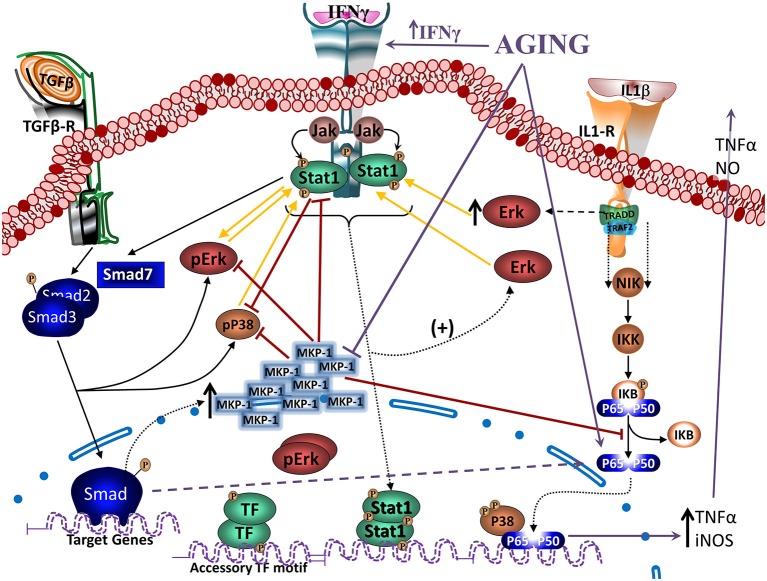
**Regulation of Janus activated kinase (JAK)-Stat and nuclear factor kappa B (NFκB) signaling by TGFβ through MKP-1.** Modulation of Interferon gamma (IFNγ)- and Interleukin 1β (IL1β)- induced signaling pathways (as models of inflammatory activation) by TGFβ1 reveals, at least partially, the anti-inflammatory effects of TGFβ1. IFNγ activates JAK- signal transducer and activator of transcription-type-1 (STAT1) pathway, increasing pSTAT1tyr, pERKs and to a lesser extent pP38. ERK and P38 MAPKs potentiate STAT1 activation by phosphorylation of a serine residue. IFNγ-induced signaling pathway inhibits Smad signaling by increasing synthesis of its endogenous inhibitor Smad7. TGFβ1 is also able to activate MAPKs. Particularly, TGFβ1 can activate ERK and P38 and also, through the activation of the Smad3 pathway, induces an increase in MKP-1 expression. Particularly in microglia, TGFβ1 inhibits IFNγ-induced STAT1 activation via a MKP-1-mediated inhibition of ERK1/2. Cytokines, such as IL1β induces activation of glial cells and the production of TNFα and nitric oxide (NO) through the activation of P38 and NFκB pathways. TGFβ1, by inducing an increase in MKP-1 levels, inhibits P38 and NFκB pathways, reducing production of TNFα and NO. In aging, there are increased levels of IFNγ, inhibiting activation of the TGFβ1-Smad signaling pathway, as well as reducing induction of MKP-1, which will result in a decreased regulation of MAPKs and NFκB pathways. An alternative pathway observed in aging leads to atypical TGFβ1 signaling that through inducing sequestering of IκB, induces activation of NFκB. Furthermore, age-related inflammation and increased production of reactive oxygen species (ROS) are strong activators of NFκB.

TGFβ-Smad pathway is very important on the regulatory and neuroprotective effect of TGFβ1 (Derynck and Zhang, 2003), being involved in the induction of the quiescent phenotype of microglia (Abutbul et al., 2012). Activation of the TGFβ1-Smad3 pathway induces glial cells to produce MKP-1, a phosphatase exerting negative regulation on inflammatory activation that inhibits Aβ-induced MAPK and NFκB signaling (Figure 4), and decreases production of TNFα and NO (Flores and von Bernhardi, 2012). MKP-1 appears to preferentially dephosphorylate P38 and JNK, but it also dephosphorylates ERK in some cell types (Liu et al., 2007; Boutros et al., 2008).

TGFβ1-dependent regulatory mechanisms are impaired in aging. Aged microglia show a basal activated status, which has been linked to neuronal damage, cognitive impairment, and an increased susceptibility to neurodegenerative diseases, such as AD (Block et al., 2007). Age-related alteration of TGFβ pathway includes changes in TGFβ release, Smad3 activation, and on microglial response induced by inflammatory stimuli in the hippocampus of aged mice, as well as abolition of TGFβ-induced phagocytosis (Tichauer and von Bernhardi, 2012) by aged microglia (Tichauer et al., 2014). In addition, Smad2/3 expression pattern is altered, showing increased expression of Smad3 in aging. In contrast, Smad2 (Deltaexon3), a splice form of Smad2 that directly binds to the DNA, is highly expressed prenatally and in early postnatal life, but it diminishes with aging (Ueberham et al., 2009).

Both age and inflammatory status affect the amount and phosphorylation of Smad3 protein in mice hippocampus (Tichauer et al., 2014). Whereas 2-month old mice show a robust increase of Smad3 in the hippocampus after a systemic inflammatory stimulus, 12-month old animals maintain Smad3 at its increased basal level (Figure 3). Similarly, phosphorylation (activation) of Smad is not induced by inflammation in old animals (Tichauer et al., 2014). The activation of the Smad pathway in young animals could depend on the effective elevation of TGFβ1 levels induced by inflammatory stimulation (Wynne et al., 2010). The induction of Smad3 expression could depend on the activation of MAPK1 (Ross et al., 2007). In contrast, in adult mice, increased basal levels of TGFβ1 (Colangelo et al., 2002; Lukiw, 2004) maintains elevated Smad3, becoming unresponsive to new inflammatory stimulation. Increased levels of TGFβ1 with a reduced activation of Smad signaling can result in an unbalance between the various TGFβ1 activated pathways (Schmierer and Hill, 2007). Furthermore, considering that the non-Smad TGFβ1 pathways MAPKs and PI3K, also participate in inflammatory activation signal transduction, and their activation is not abolished in aged mice, inhibition of Smad could abolish the regulatory effect of TGFβ1 on inflammation, facilitating the cytotoxic activation of glia. The partial inhibition of TGFβ1-Smad3 signaling in aging could explain the persistent activation of microglia and mild neuroinflammation, regardless the elevated levels of TGFβ1 in aged mice. The lost ability to modulate microglia activation, together with the increased ROS production by aged animals, could result in a predominantly cytotoxic activation.

### Age-Related Changes on TGFβ and NFκB

The transcription factor NFκB is a robust candidate for showing age-dependent changes due to its role in the regulation of immunity, inflammation, and cell death (Adler et al., 2007). Blockade of NFκB in aged mice has been reported to reverse the gene expression program and cell morphology, “rejuvenating” old mice (Adler et al., 2008). Robust evidence in a variety of cell and animal based experimental systems show that oxidative stress and inflammation are strong inducers of NFκB activation (Muriach et al., 2014). They are frequently associated with aging, and are involved in the pathophysiology of several chronic diseases observed in aged individuals, like diabetes and AD. In fact, Aβ can be a strong inducer of NFκB in neuron cell death via the induction of intracellular ROS (Lee et al., 2005b; Valerio et al., 2006), and through tumor necrosis factor receptor 1 (TNFR1) signaling, which result in neuronal apoptosis (Li et al., 2004; Valerio et al., 2006). Inhibition of these pathways could be beneficial in the treatment of neurodegenerative diseases, including AD (Lee et al., 2005a,b; Munoz et al., 2007; Paris et al., 2007; Wang et al., 2008a). The effect appears to involve attenuation of Aβ-induced activation of ERK1 and P38 MAPKs, which are upstream NFκB signaling pathway (Pannaccione et al., 2005; Valerio et al., 2006).

TGFβ1 can also promote inflammatory activity under certain conditions. Yan et al. showed that TGFβ1 injection to the hypothalamus resulted in inflammatory NFκB signaling. Activation is via the TGFβ-R2 receptors expressed on neurons of the medial basal hypothalamus. It induces formation of RNA stress granules that accelerate the decay of IκBα, resulting in activation of NFκB (Yan et al., 2014). Although the work was oriented to understanding mechanisms of diabetes, similar conditions are also observed in aging.

In addition to this novel mechanism of TGFβ-dependent activation of NFκB, aging also shows reduction of MKP-1 (see subsection “Age-Related Changes on TGFβ and MKP-1”) that impairs inhibitory regulation over NFκB and MPAKs, potentiating cell reactivity, inflammatory activation, and potentially oxidative stress and cytotoxicity. In addition, increased production of ROS and neuroinflammation will result in an independent activation of NFκB.

### Age-Related Changes on TGFβ and IFNγ

**Figure 5:**
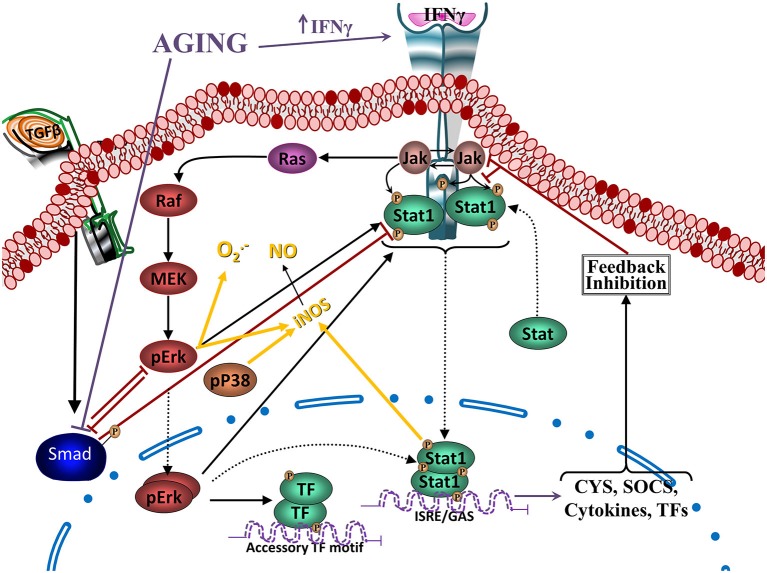
**Reciprocal regulation of TGFβ and Jak-Stat signaling.** Crosstalk between IFNγ- and TGFβ1- induced signaling pathways. IFNγ activates JAK-STAT1 pathway, increasing pSTAT1tyr, which will translocate to the nucleus and activate transcription of several cytokines and other mediators and receptors involved in inflammatory activation. In addition, it will also activate pERKs and to a lesser extent pP38. ERK and P38 MAPKs potentiate STAT1 activation and induce inducible nitric oxide synthase (iNOS), increasing production of NO, as well as production of ${\text{O}}_{2}^{-}$. IFNγ-induced signaling pathway inhibits TGFβ1-Smad signaling by inducing its endogenous inhibitor Smad7 (see Figure 4) and through inhibition by ERK. On the other hand, TGFβ1, through the activation of the Smad3 pathway, inhibits IFNγ-induced STAT1 activation via a MKP-1-mediated inhibition of ERK1/2 and iNOS expression, reducing production of NO and ${\text{O}}_{2}^{-}$. In aging, there are increased levels of IFNγ, inhibiting activation of the TGFβ1-Smad signaling pathway, as well as a reduced activation of TGFβ1-Smad, which will result in a reduced regulation of MAPKs (and NF-κB pathways as shown in the previous figure).

IFNγ is a potent activator of microglia (Ng et al., 1999; Klegeris et al., 2005). IFNγ increases in the aged brain although its endogenous cell source remains unidentified (Lyons et al., 2011). The main signaling pathways induced by IFNγ are signal transducer and activator of transcription-type-1 (STAT1) and MAPKs (Figure 5; Blanchette et al., 2003; Platanias, 2005; Gough et al., 2008). STAT1 is activated by a Janus activated kinase (JAK)-dependent phosphorylation on tyrosine Y701 (pSTAT1^tyr^) and then translocates into the nucleus to induce the expression of target genes. Full transcriptional activity requires a second phosphorylation on serine S727 (pSTAT1^ser^; Wen et al., 1995).

STAT1 is a key signaling pathway involved in the up-regulation of iNOS and NO· production (Dell’Albani et al., 2001; Gough et al., 2008). Inhibition of ERK1/2 and P38 decrease IFNγ-induced pSTAT1^ser^, which correlates with a reduction in NO· production. Decrease of pSTAT1^ser^ and NO· production is additive when both MAPK are inhibited, indicating that ERK1/2 and P38 are needed for full activation of the STAT1 pathway in glia (Figure 4), and other cell types (Blanchette et al., 2003; Platanias, 2005; Gough et al., 2008). In contrast, O_2_·^−^ production induced by IFNγ depends on increased levels of pERK1/2, but not pP38 (Bhat et al., 1998; Dang et al., 2003).

IFNγ suppresses TGFβ signaling through up-regulation of the inhibitory Smad7 (Ulloa et al., 1999), and there is a reciprocal regulatory interaction between TGFβ1 and IFNγ activated pathways (Figure 4). TGFβ1 released by hippocampal cells induce a transient increase of pERKs and a persistent increase of pP38, decreasing IFNγ-induced O_2_·^−^ and NO· production by glia (Herrera-Molina and Von Bernhardi, 2005), decreasing activation of STAT1 and ERK by IFNγ (Figure 5). Also, after persistent stimulation, IFNγ decreases TGFβ1 induced P38 signal transduction (Herrera-Molina et al., 2012). IFNγ-TGFβ1 crosstalk regulates the production of radical species through the modulation of STAT1, ERK1/2 and P38 activation. Co-treatment with TGFβ1 and IFNγ results in decreased IFNγ-induced pERK1/2, pSTAT1^ser^, pSTAT1^tyr^, total STAT1 and also reduces induction of P38 activation by TGFβ1. Suppression of pSTAT1^ser^ appears to be mediated by a TGFβ1-induced decrease of pERKs. In contrast, inhibition of pSTAT1^tyr^ depends on the decrease of total STAT1 mediated by TGFβ1 (Figure 5). Increased MKP-1 activity appears to be responsible for the reduction of IFNγ-induced activation of glia induced by TGFβ1 co-treatment. In fact, transfection with MKP-1 siRNA significantly reduces modulation of IFNγ-induced NO· production by TGFβ1. Thus, MKP-1 induction appears to be responsible of the effects on MAPK pathways and link them with those observed on the STAT1 pathway (Figure 4; Flores and von Bernhardi, 2012; Herrera-Molina et al., 2012). The regulatory interaction between TGFβ1 and IFNγ has been well described in tissue repair *in vivo*. IFNγ null mice show increased amounts of TGFβ1 and activation of TGFβ1 signaling, indicating that IFNγ exerts negative regulation of TGFβ1 activity (Ishida et al., 2004). On the other hand, TGFβ1 null mice has elevated levels of IFNγ and STAT1 activation, and iNOS and NO· production, indicating that absence of TGFβ1 results in the deregulation of IFNγ pathway and its target genes (McCartney-Francis and Wahl, 2002).

Age-related increase of IFNγ and changes on MPK-1 regulation could be key elements for the increased activation of microglia in aged animals. Aged animals show increased levels of IFNγ, directly potentiating inflammatory signaling and further inhibiting the Smad pathway through the induction of Smad7. On the other hand, decreased activation of TGFβ-Smad3 by both age-related changes and increased IFNγ, further reduce MKP-1 induction, suppressing the regulatory effect of TGFβ on inflammatory activation.

### Age-Related Changes on TGFβ and MKP-1

Among the molecular mechanism underlying the anti-inflammatory and neuroprotective effects of TGFβ1, negative regulation of MAPK signaling, key inducer of glial cell activation, is exerted by a group of MKP. In the brain, induction of MKP-1 expression in response to anti-inflammatory molecules has been demonstrated for both astrocytes and microglia (Eljaschewitsch et al., 2006; Lee et al., 2008). TGFβ1 increases MKP-1 in glia, and other cells (Jono et al., 2003; Tong and Hamel, 2007), induction that is not affected by the presence of inflammatory conditions. Increased MKP-1 reduces the activation of P38 and NFκB pathways and decreases the NO and TNFα production induced by Aβ (Flores and von Bernhardi, 2012). siRNA transfection targeting MKP-1 attenuates the effects of TGFβ1, causing a significant amelioration of the modulation of Aβ-induced TNFα and NO production by TGFβ1 (Flores and von Bernhardi, 2012). Furthermore, MKP-1 null mice show increased P38 and JNK activity and cytokine and NO production, suggesting that this phosphatase serves as an immune regulator (Liu et al., 2007; Boutros et al., 2008).

Induction of MKP-1 is mediated by the Smad3 pathway (Figure 4). Smad3 inhibition greatly reduces TGFβ1-mediated MKP-1 induction, suggesting that it is a transcriptional target for Smad3, and results in a significant amelioration of the inhibition of TNFα and NO production. MKP-1 stability and enzymatic activity can be regulated through phosphorylation and acetylation, respectively (Liu et al., 2007; Boutros et al., 2008). Although other mechanisms are involved in the regulation of the production of inflammatory mediators by TGFβ1, Smad3-mediated MKP-1 induction is a novel manner of TGFβ1 action on glia that supports its anti-inflammatory role. Increased MKP-1 levels appears also to be the mechanism of action for other anti-inflammatory molecules, such as glucocorticoids (Kassel et al., 2001; Jang et al., 2007; King et al., 2009), and it has been demonstrated that this phosphatase participates in STAT1 dephosphorylation (Venema et al., 1998).

Age-related decrease on MKP-1 secondary to the inhibition of the Smad pathway results in the impairment of the inhibitory regulation on NFκB and MAPK pathways, potentiating inflammatory activation and cytotoxicity.

### Effect of TGFβ on Stem Cells and Aging

As the brain ages, TGFβ has important roles both in neuronal survival and in the promotion of stem cell quiescence (Kandasamy et al., 2014). In the hippocampus, TGFβ appears to potentiate the survival and proliferation of intermediate progenitor cells in the dentate gyrus of aged mice, by a Smad3-dependent mechanism (Tapia-González et al., 2013). Regarding regulation of neural stem cells in the aged brain (Dias et al., 2014), TGFβ lengthens G1 phase of the cell cycle in activated stem cells, impairing cell cycle progression of neural progenitors and neurogenesis (Daynac et al., 2014). Because of those effects, blockade of TGFβ signaling could improve neurogenesis in the aged brain (Pineda et al., 2013).

Although TGFβ serves key role in neuronal surveillance and stem cell proliferation, most of the cellular changes induced by aging have been described in glial cells. Expression of TGFβ by oligodendrocytes is reduced in aging, condition that interferes with oligodendrocytes recruitment and reduces remyelination (Hinks and Franklin, 2000). In aged microglia and astrocytes, TGFβ expression shows a regional specificity. TGFβ signaling increases after brain infarct in aged individuals (Doyle et al., 2010). In contrast, microglia and astrocytes located close to the leptomeninges show reduced TGFβ expression with age, reduction that could have a role on the increased permeability of leptomeninges during systemic inflammation (Wu et al., 2008).

## TGFβ Signaling and its Role in Alzheimer Disease

There is evidence that impaired TGFβ signaling could be involved in the pathogenesis of AD. AD patients show decreased plasmatic levels of TGFβ1 (Mocali et al., 2004; Juraskova et al., 2010), but increased levels in cerebrospinal fluid (Blobe et al., 2000; Tarkowski et al., 2002), and within Aβ plaques (Burton et al., 2002). Brains of AD patients have reduced levels of TGFβRII (Tesseur et al., 2006). Reduced levels of Smad3 and impairment of Smad3 signaling have been observed in the AD brain, associated with increased Aβ accumulation, Aβ-induced neurodegeneration and neurofibrillary tangle formation (Luterman et al., 2000; Colangelo et al., 2002; Katsel et al., 2005; Tesseur et al., 2006). In addition to decreased expression of Smad3 in hippocampi of AD patients, hippocampal neurons show increased levels of activated Smad2 (Lee et al., 2006), along with alterations in the subcellular localization of phosphorylated Smad2/3 (Colangelo et al., 2002; Lee et al., 2006), which remains in the cytoplasm of neurons, instead of translocating into the nucleus (Lee et al., 2006; Ueberham et al., 2006). The ectopic localization of activated Smads in AD could be attributed to another pathological feature observed in AD, the hyperphosphorylation of tau. Hyperphosphorylated tau is associated with the sequestration of activated Smad2/3, and the disruption of TGFβ signaling (Baig et al., 2009). Both reduced presence of TGFβRII and defects on Smad are associated with inhibition of TGFβ signaling.

The TGFβ pathway exerts direct regulation over some of the pathological features of AD, since it upregulates the expression of the APP in normal human astrocytes by a Smad4-dependent mechanism (Burton et al., 2002). TGFβ also favors stabilization of the APP mRNA by binding it to a RNA-protein complex that reduces the rate of APP mRNA decay (Amara et al., 1999). In addition, TGFβ1 has been implicated on the promotion of amyloid angiopathy in frontal cortex and meninges (Wyss-Coray et al., 1997, 2000; Mazur-Kolecka et al., 2003), and on the increased production of Aβ by astrocytes in APP/TGFβ1 transgenic mice (Lesne et al., 2003). Notoriously, this amyloid angiopathy appears at a younger age when the overexpression of TGFβ1 in astrocytes occurs in the absence of SR-A (TGFβ1/SR-A^−/−^ mice) (Lifshitz et al., 2013), which lead us to further inquire on the expression of that receptor, as discussed on the next two sections. Furthermore, those effects appear to depend on the activation of astrocytes that stimulate the production of APP due to the presence of a TGFβ1 response element in the 5’UTR of APP. Moreover, Tg2576 mice with a dominant negative TGFβ1-receptor II that blocks Smad2/3 signaling show a conspicuous reduction of amyloid deposits in the brain (Town et al., 2008).

In contrast with those potentially deleterious effects of TGFβ1, increased TGFβ1 has been also associated with a lower burden of Aβ in the parenchyma, which correlates with an increased microglia activation. Several reports show that TGFβ1 has anti-amyloidogenic roles, reducing the Aβ burden and inhibiting the formation of neuritic plaques, effects that appear to be mediated by the promotion of microglia-mediated Aβ degradation (Wyss-Coray et al., 2001). The neuroprotective role of TGFβ1 against Aβ toxicity has been studied *in vitro* and *in vivo* models of AD (Prehn et al., 1996; Caraci et al., 2008). Furthermore, TGFβ1 Smad3 also inhibits the production of radical species induced by inflammatory stimuli, and induces phagocytosis of Aβ *in vitro* (Tichauer and von Bernhardi, 2012).

However, induction of phagocytosis is lost as animals age (von Bernhardi et al., 2011). Smad3 pathway is altered in microglia from adult mice, affecting the induction of Aβ phagocytosis and the modulation of radical species production by TGFβ1.

The uncoupling of TGFβ1 signal transduction pathway could result in an altered pattern of microglial activation and reduced clearance of amyloid; effects that in fact are observed in aging and in AD. Impairment of TGFβ signaling can potentiate neuroinflammation, favoring neuronal dysfunction and neurodegenerative changes (Tesseur and Wyss-Coray, 2006). Reduced TGFβ-Smad3 signaling results in age-related neuroinflammation and neurodegeneration and in increased accumulation of Aβ (Tesseur et al., 2006). The accumulation of Aβ could depend on a reduction of its clearance, and be mediated by the reduced expression of SR-A by glia (Tichauer and von Bernhardi, 2012). These changes could facilitate cytotoxic inflammation and neurodegenerative diseases in aging (von Bernhardi et al., 2011). If accumulation of Aβ depends indeed on impaired clearance, it could situate Aβ as the result of disease progression instead of being its primary cause, as we propose in the “microglial cell dysregulation” hypothesis for AD.

## TGFβ Regulates SR-A

The effect of TGFβ is cell and tissue specific. Whereas TGFβ reduces expression of some SRs in circulating macrophages, TGFβ increases expression of SR-A, while decreases expression of SR-BI by microglia (Tichauer and von Bernhardi, 2012). Given the relevance of SRs as well as other pattern recognition receptors (PRRs) on the inflammatory activation and the scavenger function of microglia, changes on the expression of these receptors could have a profound effect on microglial cell activation (von Bernhardi, 2007).

In addition to phagocytosis, SR-A is also involved in the regulation of glia activation (Murgas et al., 2014). Accordingly, the use of SR-A antagonists appears to improve the phenotypic features of AD (Handattu et al., 2009) by reducing microglial activation (Handattu et al., 2013). These results support the idea that SR-A activity could be part of the molecular mechanism involved in glial cell activation. In contrast to most SRs, SR-A expression is not necessarily downregulated, but can be increased by its ligands (Nikolic et al., 2011). In addition, binding of ligands to SRA recruits SRs to the membrane surface by a PI3K-activated mechanism (Cholewa et al., 2010). In macrophage cell lines, like THP-1 and J774A.1 (Bottalico et al., 1991; Nishimura et al., 1998; Draude and Lorenz, 2000; Argmann et al., 2001; Michael et al., 2012) and human monocytes (Draude and Lorenz, 2000), TGFβ1 reduces SR-A expression, through a mechanism that depends on Smad-2 (Michael et al., 2012). In addition, it has been also reported that induction of TGFβ1 by statin treatment, abolish induction of SR-A by inflammatory stimuli, in a mechanism mediated by ERK activation (Baccante et al., 2004).

In addition of increasing their expression of SR-A, TGFβ1 also increases Aβ uptake by microglia, an effect that is prevented by the Smad3 inhibitor SIS3 (Tichauer and von Bernhardi, 2012). Studies by our laboratory have also shown that induction of Aβ phagocytosis by TGFβ1 is decreased in aged mice (Tichauer et al., 2014). Reduction of induction of Aβ phagocytosis, together with decreased expression of SR-A in the brain of aged APP/PS1 mice (Hickman et al., 2008), suggest the existence of an altered regulation of SR-A expression in aging. Given its participation in Aβ uptake, SR-A impairment could be involved in the accumulation of Aβ during aging, with a mechanism associated with TGFβ1-Smad3 signaling (Tichauer and von Bernhardi, 2012), and could be related with AD pathogenesis.

## Gene Transcription Regulation by TGFβ SR-A as a Model

**Figure 6:**
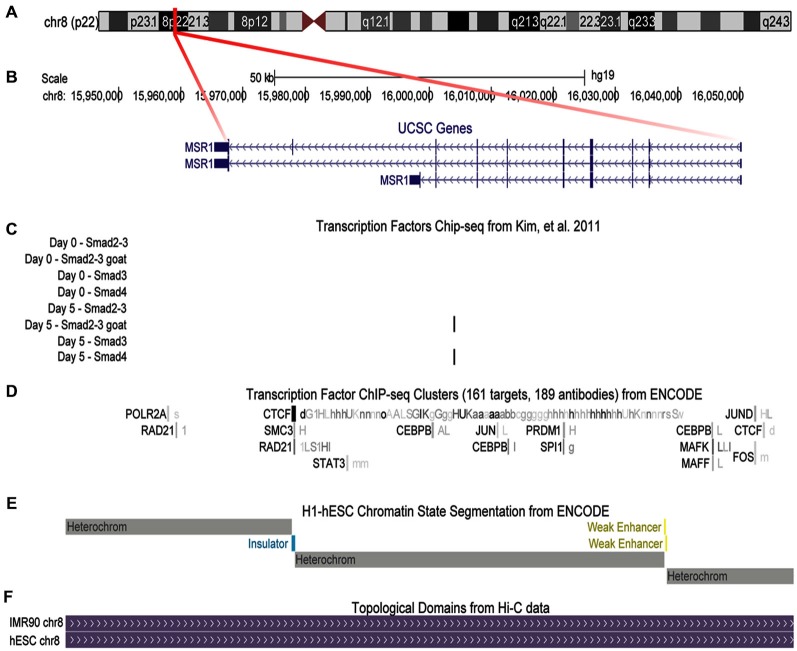
**Binding of transcriptional co-factors Smad2/3 and 4 to the MSR1 gene.** SR-A gene structure is shown using UCSC gene annotation, ChIP-Seq available data, and chromatin structure associated to this gene. **(A)** Ideogram of chromosome 8. The region labeled in red shows the MSR1 gene location. **(B)** MSR1 gene structure, indicating the three most common isoforms. Arrows indicate the direction of transcription and boxes symbolize exons. **(C)** ChIP-Seq data for Smads using four different antibodies before activin treatment (day 0) and 5 days after treatment of human embryonic stem cells (hESC; Kim et al., 2011); black bars show significant signals of ChIP-Seq indicating Smad2/3 and Smad 4 binding to MSR1 gene after activin treatment (day 5). **(D)** ChIP-Seq results provided by ENCODE project for 161 transcription factors from various cell lines. Boxes represent ChIP-Seq significant signal for a specific transcription factor. The darkness of the box is proportional to the maximal signal intensity of ChIP-Seq observed in a cell line, shown next to the box, in a lowercase letter, cells where this signal was found significant. **(E)** Chromatin segmentation state data generated by ENCODE project for H1-hESC (Aad et al., 2011); gray boxes represent heterochromatin sites, yellow is used for enhancer sites, and blue are for insulators. **(F)** Genomic topological domains detected by using Hi-C data in hESC and IMR90 cell lines (Dixon et al., 2012).

Given our observation of increased protein expression of SR-A in microglia exposed to TGFβ through a Smad3-dependent mechanism (Tichauer et al., 2014), we performed *in silico* analyses of human genomic data to determine if the effect could be mediated by the transcriptional co-factors Smad2/3 over the MSR1 gene coding SR-A. As shown in Figure 6A, MSR1 gene is located at chromosome 8 and its transcription commonly generates three main splicing variants (Figure 6B). Analysis of ChIP-Seq data generated by Kim et al. revealed a significant peak of binding of Smad2/3 and Smad4 at the intron 7 of the MSR1 gene when human embryonic stem cells (hESC) were treated with activin (Figure 6C), a TGFβ receptor agonist (Kim et al., 2011). In addition, the MSR1 gene is ubiquitously bound by CTCF (Figure 6D), a protein factor having a key role in chromatin topological regulation (Ong and Corces, 2014) and previously described as a robust transcriptional repressor (Holwerda and De Laat, 2013) in various cell lines. The chromatin state segmentation data shows that the CTCF binding site is located in a genomic insulator region that is surrounded by highly repressed chromatin (Figure 6E), and is correlated with the presence of a topological domain predicted from Hi-C experiments (Figure 6F; Dixon et al., 2012). The presence of a CTCF peak in the MSR1 gene and the fact this gene is in a topologic domain suggest that MSR1 is highly repressed in most cell types (Figures 6D–F). This could explain the tissue specificity of this gene, which is almost exclusively present on the monocyte-macrophage lineage (Christie et al., 1996; Godoy et al., 2012), and astrocytes (Alarcón et al., 2005).

**Figure 7:**
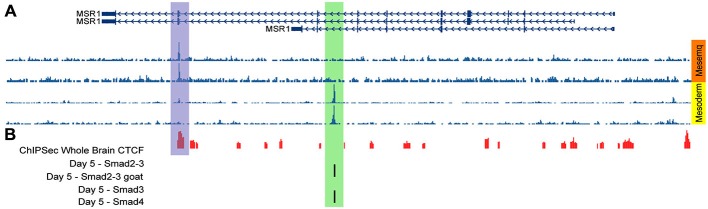
**DNase sensitive sites matching with CTCF binding sites after activin treatment. (A)** DNase hypersensitivity data in H1-hESC cells differentiated into mesenchymal cells (orange block) and treated with activin to differentiate H1-hESC cells into mesoderm (yellow block); different rows correspond to different experiments. **(B)** ChIP-Seq data for CTCF in the whole brain (red bars) and for Smad 2/3 and 4 (black bars; Kim et al., 2011).

We also analyzed data of DNase sensitivity and ChIP-Seq available at WashU Epigenome Browser (Zhou et al., 2013), finding that H1-hESC cells differentiated into mesenchymatic cells present a hypersensitivity peak to DNase coincidental to the CTCF binding site (Figure 7A). In contrast, after treatment of H1-hESC cells with activin to differentiate them into mesoderm, the DNase hyper-sensitivity peak related to CTCF disappears, but a new DNase hypersensitivity peak is observed, which match with the Smad2/3 and Smad 4 binding site detected by the previous ChIP-Seq data analysis (Figures 7A,B; Kim et al., 2011). This shift between those two DNase hypersensitivity peaks after activin treatment leads us to infer that TGFβ pathway activation interferes with the binding of CTCF and might promote a topological rearrangement that induces MSR1 transcription by a mechanism mediated by Smad2/3/4. This hypothesis is currently been experimentally tested by our group.

The brain of AD patients reveal changes on the methylation pattern of some genes described as susceptible to be involved in AD, such as PSEN1 y APOE, and in genes related to homeostasis of gene methylation, like MTHRF and DNMT1 (Wang et al., 2008b). This study also identified age-specific epigenetic changes, suggesting that epigenetics could have a role in the development of AD (Wang et al., 2008b). Given that possibility, a future challenge is to assess the existence of epigenetic changes on the MSR1 gene or on genes related with the TGFβ pathway during aging and in AD. Changes potentially affecting SR-A expression or function as the brain ages would affect inflammatory activation and Aβ uptake by glia.

## Concluding Remarks

Age dependent changes such as microglia mis-activation, production of ROS, and decreased proteasome activity could establish the grounds for microglial cell dysfunction, leading to cytotoxicity and accumulation of Aβ or other protein aggregates. The combined effect of various age-associated changes, in addition to the individual endophenotypic condition and diverse environmental stimuli can initiate the vicious circle of cytotoxic activation of microglia.

Innate immune response, with microglia as the pivotal player, is recognized to have a profound immune-modulatory and reparative potential. However, chronic activation and dysregulation of microglia can lead to deleterious effects, inducing malfunction and damage of the CNS. Microglia activation appears to undergo different phases depending on their environmental and functional context. Whereas inhibition of microglia can be beneficial at a certain phase of disease progression it can become detrimental at another. A critical area of research would be to understand their activation process, developing pharmacologic tools directed towards selected properties of microglia. That would be a major improvement respect the present approach of turning off microglial cell activation as a whole, which likely has a major bearing in the limitations of past thinking about immunoinhibitory drugs for neurodegenerative diseases.

**Figure 8:**
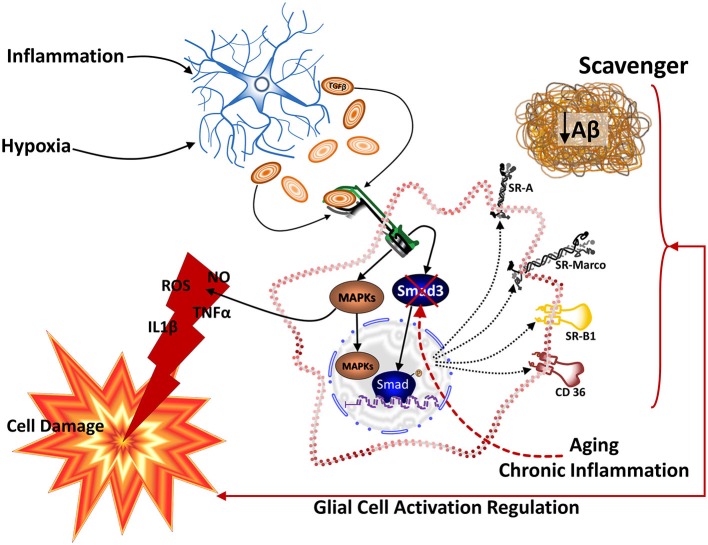
**Model for TGFβ1-Smad3 pathway regulation in aged microglia.** Diverse stimuli, including inflammatory stimulation and hypoxia, induce astrocytes to secrete TGFβ1. Binding of TGFβ1 to its receptor results in the activation of the Smad3 pathway, as well as MAPKs and PI3K signaling in microglia. Thus, TGFβ1 regulates the inflammatory activation of microglia in addition to modifying the SRs profile expressed by the cell. SRs appear to be involved both in the uptake of Aβ and in the activation of glial cells. Several of the effects of TGFβ1 on cell viability, reduction of inflammatory cytokines and reactive species, and expression of SRs depend on the activation of Smad3. In aging or after exposure to chronic inflammatory conditions, canonical activation of Smad3 is greatly reduced, whereas MAPKs remains activatible. As result of this change on TGFβ1 signaling, microglia show increased cytotoxicity, undergo changes on their expression of SRs and decrease their Aβ clearance. Thus, reduced TGFβ1-Smad3 activity on aged microglia appears to impair the beneficial effect of TGFβ1.

Our interest in identifying protective and regulatory pathways, to potentiate them while inhibiting cytotoxic activation of microglia, lead us to study the effect of TGFβ on microglia function in aging and various inflammatory conditions. TGFβ-Smad3 is involved in many protective functions of microglia, and shows major changes with aging. Our working model (Figure 8) shows that upon activation by various stimuli, TGFβ binds to its receptor activating a complex signaling pathways that includes activation of both the canonical Smad pathway as well as the non-canonical MAPKs and PI3K. As result of the activation of those pathways, among many changes, there will be changes on the expression pattern of SRs, the phagocytic activity and the production of inflammatory cytokines and other inflammatory and oxidative stress mediators. In aged microglia, increased amounts of TGFβ will act upon its receptor. However, secondary to age-related changes or chronic inflammation, the activation of Smad3 pathway is inhibited. Inhibition of Smad3 activation in the context of increased TGFβ levels shifts the regulatory signaling towards a dysregulated inflammatory activation, potentially leading to the impairment of protective response, development of an increased cytotoxicity and to neurodegenerative changes. Thus, increased neuroinflammation, decreased Aβ clearance and impaired cell viability could be consequence of the impaired TGFβ signaling.

## Conflict of Interest Statement

The authors declare that the research was conducted in the absence of any commercial or financial relationships that could be construed as a potential conflict of interest.
